# Pixel-Level and Robust Vibration Source Sensing in High-Frame-Rate Video Analysis

**DOI:** 10.3390/s16111842

**Published:** 2016-11-02

**Authors:** Mingjun Jiang, Tadayoshi Aoyama, Takeshi Takaki, Idaku Ishii

**Affiliations:** Department of System Cybernetics, Hiroshima University, 1-4-1 Kagamiyama, Higashi-Hiroshima, Hiroshima 739-8527, Japan; aoyama@robotics.hiroshima-u.ac.jp (T.A.); takaki@robotics.hiroshima-u.ac.jp (T.T.); iishii@robotics.hiroshima-u.ac.jp (I.I.)

**Keywords:** high-frame-rate video, vibration source localization, pixel-level digital filters, object tracking, drone tracking

## Abstract

We investigate the effect of appearance variations on the detectability of vibration feature extraction with pixel-level digital filters for high-frame-rate videos. In particular, we consider robust vibrating object tracking, which is clearly different from conventional appearance-based object tracking with spatial pattern recognition in a high-quality image region of a certain size. For 512 × 512 videos of a rotating fan located at different positions and orientations and captured at 2000 frames per second with different lens settings, we verify how many pixels are extracted as vibrating regions with pixel-level digital filters. The effectiveness of dynamics-based vibration features is demonstrated by examining the robustness against changes in aperture size and the focal condition of the camera lens, the apparent size and orientation of the object being tracked, and its rotational frequency, as well as complexities and movements of background scenes. Tracking experiments for a flying multicopter with rotating propellers are also described to verify the robustness of localization under complex imaging conditions in outside scenarios.

## 1. Introduction

Tracking the same object robustly against complex appearance variations is a significant task in the field of robot vision [1]. Many researchers have developed object tracking methods and systems that provide a visual representation to robustly describe the spatiotemporal characteristics of object appearance [2]. Object tracking methods using a global visual representation that reflects the global statistical characteristics of an image region to be tracked have been proposed on the basis of various global image features such as optical flows [3,4,5], color histograms [6,7,8], and texture histograms [9,10,11]. By encoding the object appearance information from the selected interest points in images, local-feature-based object tracking methods have also been proposed on the basis of local features such as scale invariant feature transform (SIFT) [12,13], Haar-like features [14,15], the histogram of oriented gradient (HOG) [16,17,18], and the local binary pattern (LBP) [19,20,21]. These appearance-based object tracking methods have been applied in various real-world applications such as traffic monitoring [22,23,24], video compression [25], and human-computer interaction [26,27].

Several unsupervised and semi-supervised object detection methods have been recently proposed to improve the localization accuracy in object tracking. These methods are based on spatio-temporal appearance cues across video frames such as max-path search [28,29], tubelets [30], fast proposal [31], action tubes [32], bag of fragments [33], and stacked sequential learning (SSL) [34].

However, most appearance-based approaches assume that the target object is being tracked by identifying its spatial statistical pattern, and that the target object can be observed in a certain image region in which its spatial distribution represents its visual appearance. Several attempts at tracking low-resolution targets have been developed [35,36]. However, appearance-based object tracking suffers from difficulties in handling complex real-world changes in object appearance, which are caused by factors such as illumination variation, lens defocus, shape deformation, and partial occlusion.

Many real-time, high-speed vision systems that can process images at 1000 frames per second (fps) or more have been developed [37,38,39,40]. Our research group has demonstrated their effectiveness by implementing object tracking algorithms such as optical flow estimation [41], color-histogram-based tracking [42], corner-point-based image mosaicing [43], Haar-like-feature-based face tracking [44], and depth-image tracking [45]. Several high-speed vision systems have been used in applications such as high-speed mechanical tracking [46], high-speed grasping of a robot hand [47], and virtual stillness for beating heart surgery [48]. Dynamic sensing applications for phenomena that are unmeasurable by the human eye and standard cameras operating at dozens of frames per second have also been developed, such as laboratory animal scratching behavior analysis [49], microchannel-based cell deformation analysis [50,51], and vision-based modal analysis [52].

State-of-the-art methods and application systems using real-time high-speed vision have been reviewed in [53]. Assuming that the vision system has a sufficiently high frame rate for vibration measurement, offline high-frame-rate (HFR) video analyses have been also reported as optical sensing methods for audio frequency level vibration distributions, and they have been applied to various applications, such as structural vibration analysis [54,55,56] and human vocal fold vibration [57,58,59]. Ishii et al. proposed a vision-based vibration source tracking method that can detect the temporal periodic changes in image intensity at pixels around vibration sources by implementing digital filters at all the pixels. These are the basic operations in acoustic signal processing for the analysis of sound and vibration dynamics, and were used in real-time target tracking experiments for a vibrating object [60]. For sound source localization, numerous acoustic-signal-processing methodologies have been proposed for applications such as robot auditory systems [61] and industrial applications [62]. Recently, several audio-signal-based drone surveillance systems have been developed [63,64,65,66], because the detection of illegal or abnormal objects is a growing concern following the recent popularization of flying drones. However, the localization accuracy of these acoustic methods remains limited, because of the low directivity in sound propagation. If the periodic brightness changes at pixels around the flying drone could be extracted from images, the vibration-based object tracking approach would be more accurate, allowing robust drone localization and tracking even under complex changes in the drone’s appearance in the real environment.

Therefore, in this study, we verify the robustness and pixel-wise accuracy of localization against several appearance variations in the vibration-based object tracking approach by analyzing the periodic brightness changes in the audio frequency range in HFR videos. The remainder of this paper is organized as follows. Section 2 describes the vibration feature with pixel-level digital filters [60], which should be evaluated as pixel-wise vibration features for tracking vibrating objects. Section 3 presents several 2000 fps video analysis results for detecting the periodic brightness changes around a rotating fan under variations in brightness, defocus blur, apparent scale with distance, pose orientation, and rotational frequency, as well as under complex and moving background scenes. Section 4 describes the experimental results from tracking a multicopter whose propellers are rotating at 80–100 Hz in outside scenarios with complex imaging conditions. It is shown that robust tracking can be achieved even when the image region of the drone is of low resolution or low quality.

## 2. Vibration Feature with Pixel-Level Digital Filters

**Figure 1:**
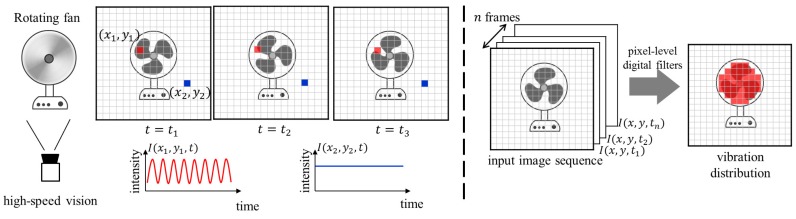
Concept of vibration features with pixel-level digital filters.

An image sensor can be regarded as a collection of photo sensors, and the image intensity at every pixel can be considered as a time sequential signal for temporal brightness variations. When the target object has a specific visual vibration, the vibration-based object tracking approach on the basis of dynamics-based features at every pixel enables the accurate localization of the target object, depending on the acute directivity of light propagation. And it also enables robust vibration source localization against complex object visual appearance changes, because of the unique dynamics properties of vibration sources. This is clearly different from conventional appearance-based tracking methods with spatial pattern recognition. Figure 1 illustrates the concept of the vibration-based object tracking [60] evaluated in this study, wherein image features are calculated from vibration distributions using pixel-level digital filters that can identify a certain frequency component in the brightness variation at each pixel. Assuming that the input image of N×N pixels is captured at time *t* as I(x,t), and the properties of a vibrating object are initially given, such as its center frequency $f_{0}$. The vibration feature to be evaluated in this study is calculated as follows:

(1) Pixel-level band-pass filter

The input image I(x,t) is filtered at every pixel x=(x,y) with a band-pass filter of the center frequency $f_{0}$ by adopting the following infinite impulse response (IIR) filter:

(1) $$g\left(x,t\right)=\sum_{s=0}^{p-1}b_{s}I\left(x,t-s\right)-\sum_{s=1}^{p-1}a_{s}g\left(x,t-s\right)$$

where *p* is the filter order and $a_{s},b_{s}$ are the tap coefficients. These parameters determine the center frequency and bandwidth of the filter.

(2) Amplitudes of filtered image intensities

To remove the offset values in the image intensities, the differences between the maximum and minimum values of I(x,t) and g(x,t) are computed at every pixel over a cycle of the target’s vibration, $T_{0}=1/f_{0}$, for $t-T_{0}$ ∼ *t* as the following amplitudes of the image intensities at time *t*:

(2) $$I_{A}\left(x,t\right)\;=\;I_{max}\left(x,t\right)-I_{min}\left(x,t\right)$$

(3) $$g_{A}\left(x,t\right)\;=\;g_{max}\left(x,t\right)-g_{min}\left(x,t\right)$$

where the maximum and minimum values are calculated as follows:

(4) $$I_{max}\left(x,t\right)=\max_{t-T_{0}<t^{\prime }\leq t}I\left(x,t^{\prime }\right)\;I_{min}\left(x,t\right)=\min_{t-T_{0}<t^{\prime }\leq t}I\left(x,t^{\prime }\right)$$

(5) $$g_{max}\left(x,t\right)=\max_{t-T_{0}<t^{\prime }\leq t}g\left(x,t^{\prime }\right)\;g_{min}\left(x,t\right)=\min_{t-T_{0}<t^{\prime }\leq t}g\left(x,t^{\prime }\right)$$

(3) Moving averages of filtered amplitudes

The average amplitude value of the brightness of the input image in a certain interval $\Delta T_{f}$ and that of the intensity and the filtered image are calculated at every pixel as:

(6) $$K(x,t)\;=\;\frac{1}{\Delta T_{f}}\int_{t-\Delta T_{f}}^{t}I_{A}\left(x,t\right)dt$$

(7) $$G(x,t)\;=\;\frac{1}{\Delta T_{f}}\int_{t-\Delta T_{f}}^{t}g_{A}\left(x,t\right)dt$$

where $\Delta T_{f}$ is set to several times the cycle time $T_{0}$.

(4) Vibration pixel localization

By thresholding the ratio of G(x,t) to K(x,t) with a threshold $\theta_{2}$, the pixel x is judged to be a vibration pixel with the vibration component around the target frequency $f_{0}$ as follows:

(8) $$V\left(x,t\right)=\left\{\begin{array}{cc} 1 & \left(K\left(x,t\right)>\theta_{1}\;\mathrm{and}\;\frac{G(x,t)}{K(x,t)}>\theta_{2}\right)\\ 0 & \left(\mathrm{otherwise}\right) \end{array}\right.$$

where the pixel x is judged to be ambiguous and not extracted when the average amplitude G(x,t) is lower than a threshold $\theta_{1}$.

Such a vibration feature can detect the temporal brightness variation in the audio-frequency range at every pixel on the premise that the input images are captured at a high frame rate.

**Figure 2:**
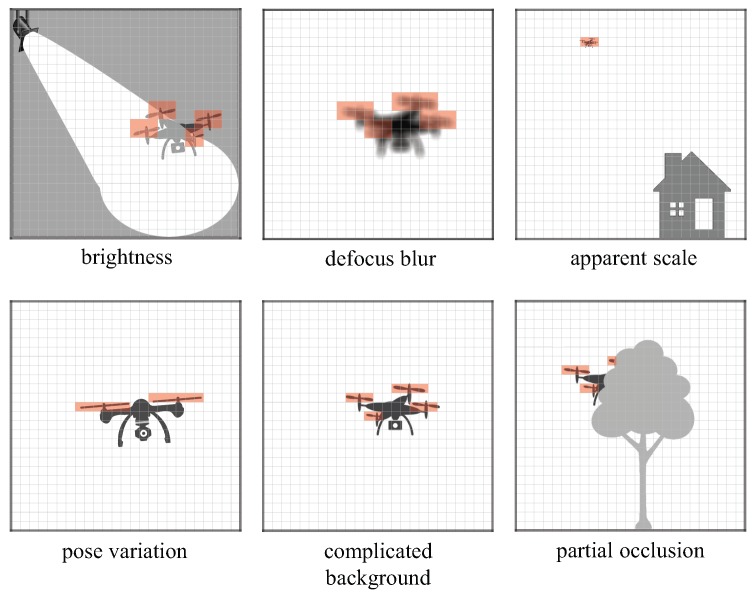
Robustness of vibration features with pixel-level digital filters.

Thus, it is very robust against the degradation of the image quality and the target’s appearance variation especially when the frequency range of the vibration source is largely distant from that of background scenes, as illustrated in Figure 2, because it enables pixel-wise vibration source localization only by implementing band-pass filters at all the pixels in images without any spatial appearance representation. Such a very simple vibration feature with band-pass filters is suitable for real-time vibration source localization for drone tracking, where the operation frequency range of the drone’s propellers is much higher than that of the temporal brightness changes at pixels around non-propeller regions in images. When a vibrating object such as a flying drone with rotating propellers is captured in low-quality images using a zoom camera at a very-long distance (and thus with limitations on the resolution of the lens and image sensor), the pixel-wise vibration feature can accurately localize the vibrating object in the low-quality images. This is despite the images being too spatially defocused or low-resolution for conventional appearance-based approaches to identify the target. Thus, in the design of vibration-object tracking systems, it is important to quantitatively verify the localization accuracy and detectability of such a pixel-wise vibration-feature under degraded video-shooting conditions (such as poor brightness, lens defocus, and low-resolution images) and confirm its robustness against object appearance variations (such as object pose variations, complex background scenes, and partial occlusions).

In this study, we focus on offline quantitative verification of the accuracy and detectability in localizing a vibration source such as a flying drone with rotating propellers by using HFR videos, whereas we evaluate the execution times of our algorithm on a personal computer (PC) in calculating the above-mentioned processes of (1)∼(4) toward future real-time implementation. Table 1 summarizes the execution times for our algorithm for different image sizes. Here we used a PC with an ASUSTek SABERTOOTH X79 mainboard, Intel Core i7-4820K @ 3.70 GHz CPU, 8GB memory, and two 16-lane PCI-e 2.0 buses with Windows 7 Enterprise 64-bit OS, and the filter order was set to p=4, which is the same parameter used in the experiments in Section 3 and Section 4. The execution time for our algorithm increased in proportion with the total number of image pixels. In the case of real-time software execution, the operable frame rates of a vision system are 6143, 1517, 372, 96, 25, and 6 fps for images with different sizes of 64 × 64, 128 × 128, 256 × 256, 512 × 512, 1024 × 1024, and 2048 × 2048 pixels, respectively. Low resolution images can only be processed by software in real time at thousands of fps, whereas our algorithm should be accelerated for real-time processing of higher resolution images at high frame rates by implementing parallel processing logics of our algorithm on specific accelerators such as FPGAs (Field Programmable Gate Arrays) and GPGPUs (General-Purpose Graphic Processing Units).

## 3. Experiments for a Rotating Fan

We extracted the vibration features from high-frame-rate videos captured with different lens settings to consider the robustness under the following seven imaging conditions.

### 3.1. Image Intensity

Several 512 × 512 videos of a rotating fan were captured at 2000 fps with different aperture values, which were adjusted to simulate various image intensities. We applied pixel-level digital filters to these videos to analyze the robustness of the proposed vibration-based localization method under brightness variations.

**Figure 3:**
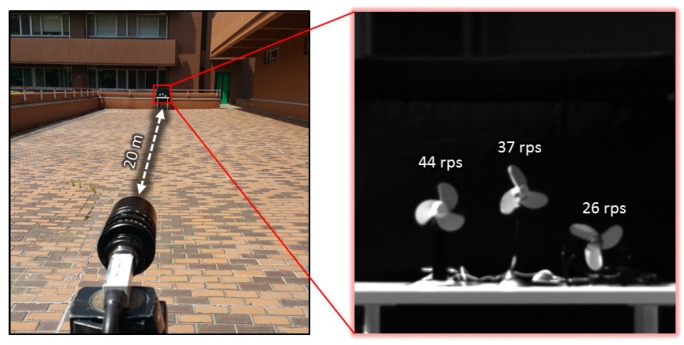
Overview of high-frame-rate video shoot.

Figure 3 illustrates the video shooting conditions. Three 13-cm-diameter fans with three blades were set at a distance of 20 m in front of the camera against a black background. The center fan was the target, rotating at 37 revolutions per second (rps), and the other two fans were rotating at 44 rps and 26 rps (left and right of the camera view, respectively). These acted as obstacles to the tracked vibration motion. We used a zoom lens with an adjustable focal length and maximum aperture of 16∼160 mm and F2.0, respectively. We fixed the focal length to 90 mm, giving a measurement area of 1600 × 1600 mm for 512 × 512 pixels at a distance of 15 m in front of the camera head, where one pixel corresponds to 3.1 mm${}^{2}$. The tap coefficients $a_{s}$, $b_{s}$ of the pixel-level digital filters were set to operate as band-pass filters with center frequencies of $f_{0}=$ 110 Hz and half-widths of 10 Hz. The parameters were set to p=4, $\Delta T_{f}=$ 36 m·s, and $T_{0}=1/f_{0}=9$ m·s. The thresholds $\theta_{1}$ and $\theta_{2}$ for vibration region extraction were set to 30 and 0.5, respectively. These parameters were also used in the experiments reported in the rest of this section.

**Figure 4:**
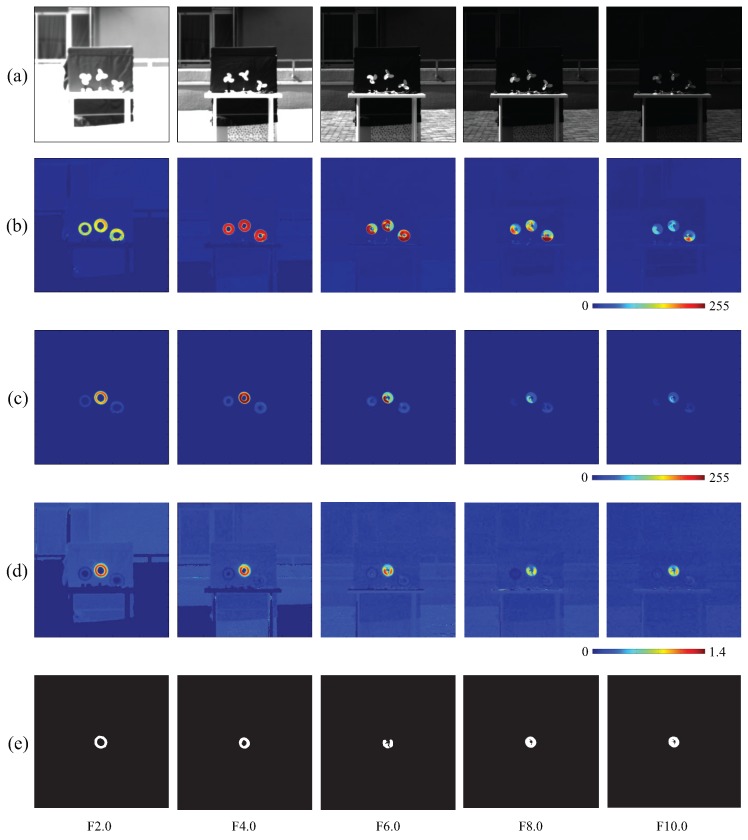
(**a**) Input images; amplitude of (**b**) input; and (**c**) pixel-wise filtered images; (**d**) amplitude ratios; (**e**) extra-cted vibration features.

The aperture value was gradually adjusted from F2.0 to F10.0 with a properly varying interval to darken the images. Figure 4a shows five input images of 512 × 512 pixels illustrating the tendency of darkening. Figure 4b,c show the moving average distributions of the amplitude of the input images and pixel-wise filtered images, respectively. With the weakening of the image intensity, the amplitude of both the input images and filtered images decreased in the vibration area. However, in Figure 4d, the amplitude ratio distributions of filtered images to input images remain roughly uniform under variations in image intensity. The vibration regions were steadily extracted by thresholding these ratio values in our proposed algorithm, as shown in Figure 4e.

**Figure 5:**
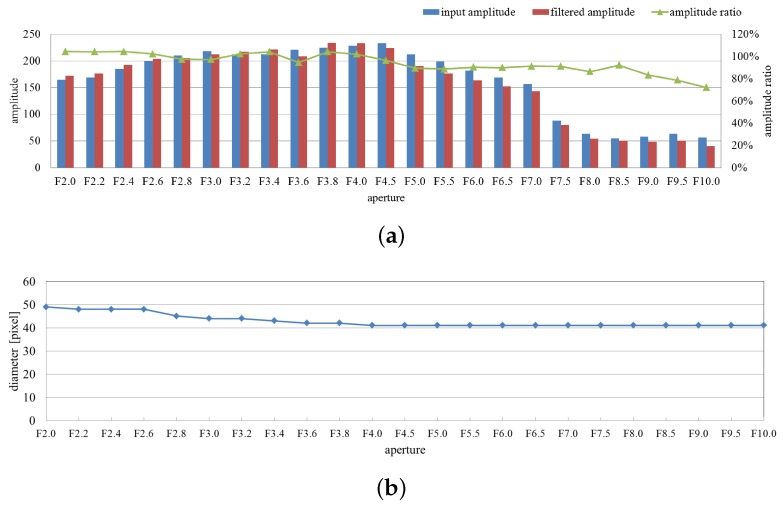
Averaged amplitudes and extracted region sizes with aperture variation. (**a**) Averaged amplitudes of input and pixel-wise filtered images and their ratios on the extracted pixels; (**b**) diameters of extracted vibration region.

The averaged values of the input and filtered images’ amplitude and their ratio in the extracted pixels are shown in Figure 5a. The diameters of the extracted vibration region are shown in Figure 5b From these figures, we can observe that, although the two amplitudes changed under image intensity variations, the ratios remained between 80% and 110%, and the diameters of the extracted vibration region corresponded to the size of the fan in the captured images (except for exceptional cases containing oversaturated images).

### 3.2. Defocus Blur

To analyze the robustness of the proposed vibration extraction method when the vibration source is out of focus, we captured several 512 × 512 videos of three rotating fans at 2000 fps with different focus distances. The three fans and their rotation speeds were as described in Section 3.1. In this experiment, they were located 5 m in front of the camera lens. The focal length and aperture value were fixed at 50 mm and F6.0, respectively. For such settings, the measurement area was 790 × 790 mm for 512 × 512 pixels at a distance of 5 m in front of the camera head, where one pixel corresponds to 1.5 mm${}^{2}$. The focus distance was gradually extended from 1.5 m to an infinite distance by adjusting the lens setting.

**Figure 6:**
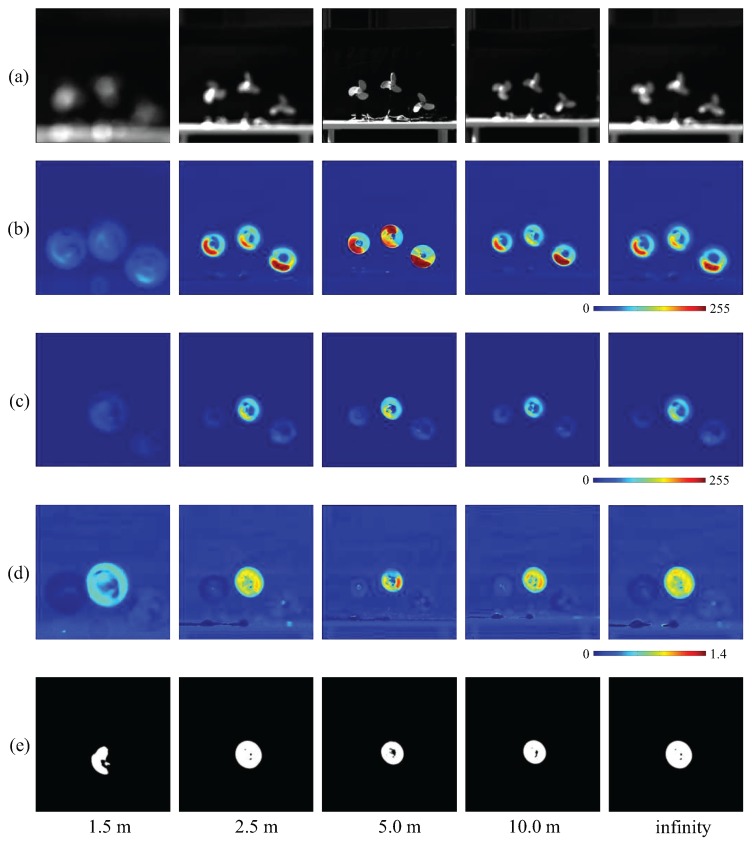
(**a**) Input images; amplitude of (**b**) input; and (**c**) pixel-wise filtered images; (**d**) amplitude ratios; (**e**) extracted vibration features.

Figure 6a shows the 512 × 512 input images contaminated by blur of different intensities. Figure 6b,c show the moving average distributions of the amplitude of input images and pixel-wise filtered images, respectively. In both cases, the amplitudes on the extracted pixels became greater when the focus distance was set around the camera-object distance and vice versa. As shown in Figure 6d, the ratio distributions of the input to filtered amplitudes on the extracted pixels remained roughly uniform at different focus distances, and these were utilized to extract clean vibration regions in Figure 6e.

**Figure 7:**
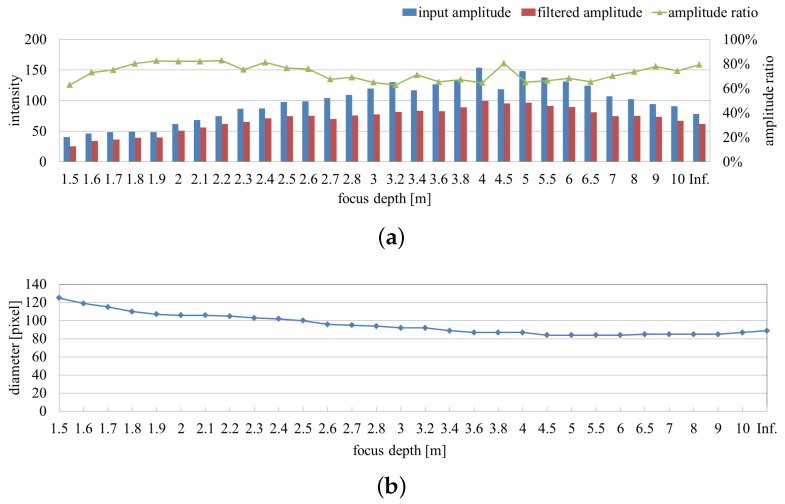
Averaged amplitudes and extracted region sizes with focus distance variation. (**a**) Averaged amplitudes of input and pixel-wise filtered images and their ratios on the extracted pixels; (**b**) diameters of extracted vibration region.

The averages of the input and filtered amplitude and their ratio on the extracted pixels are shown in Figure 7a, and the diameters of the extracted vibration region is shown in Figure 7b. From these figures, we can observe that, although the two amplitudes change significantly with variations in the focus distance, the ratio values remained between 70% and 80%. The diameters of the extracted vibration region correspond to the size of the fan in the captured images when the focus depth was set around the camera-object distance, and increased when the images were contaminated by the lens blur.

### 3.3. Apparent Scale

To analyze the robustness of the proposed vibration extraction method when the vibration source is located sufficiently remotely that it is difficult to recognize its appearance from images, we captured several 512 × 512 videos of rotating fans at 2000 fps with different focal lengths. The overall arrangement, including the camera, three fans, and their rotating speed and background, was the same as described in Section 3.1, i.e., the distance from the camera to the object was 20 m. The lens aperture was fixed to F5.0 and its focus distance was adjusted to give perfect focus. We gradually adjusted the focal length from 20 mm to 160 mm to simulate changes in the vibration source’s apparent scale in the images.

**Figure 8:**
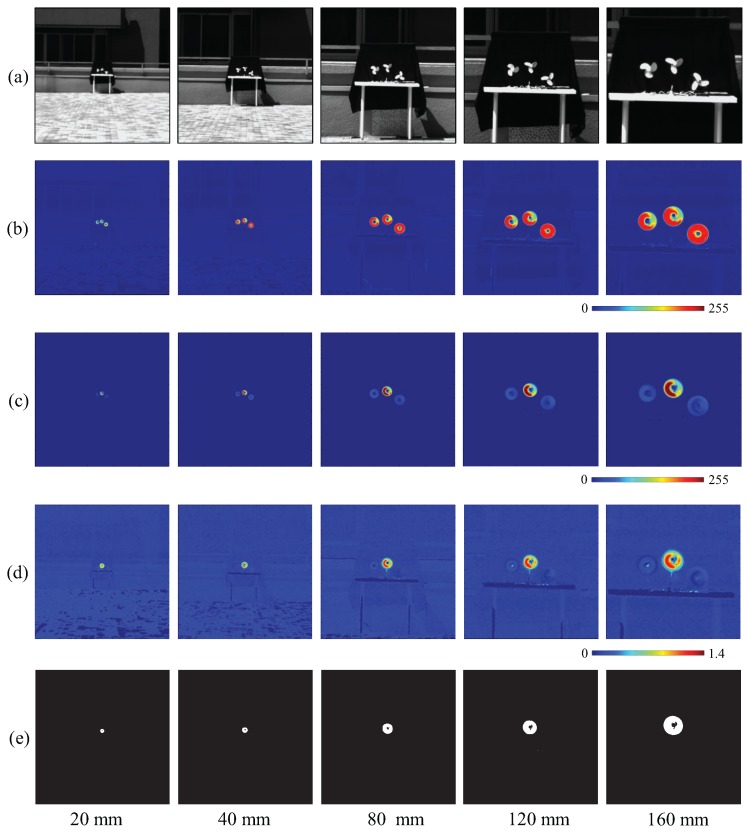
(**a**) Input images; amplitude of (**b**) input; and (**c**) pixel-wise filtered images; (**d**) amplitude ratios; (**e**) extracted vibration features.

Figure 8a shows the input 512 × 512 images of three rotating fans, whose apparent scale is increasing with the focal length. Figure 8b,c illustrate the moving average distributions of the amplitude of the input and pixel-wise filtered images, respectively. Although the two amplitudes differed while the focal length was increasing, the ratio distributions remained similar (see Figure 8d). Figure 8e shows the extracted regions given by thresholding the amplitude ratio of every pixel.

**Figure 9:**
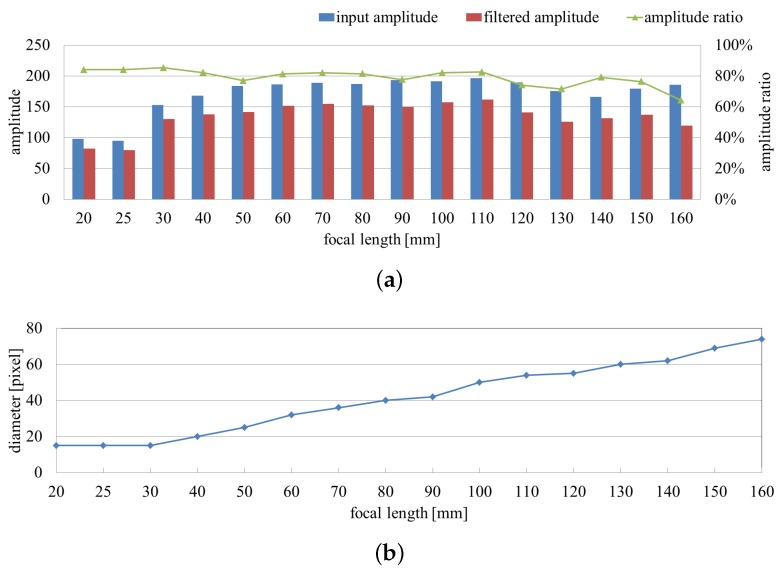
Averaged amplitudes and extracted region sizes with focal length variation. (**a**) Averaged amplitudes of input and pixel-wise filtered images and their ratios on the extracted pixels; (**b**) diameters of extracted vibration region.

Figure 9a quantifies the tendency of the averaged input and filtered images’ amplitude and their ratio distribution on the extracted pixels throughout the image-capture procedure. Although the two amplitudes change significantly, the ratio values remained around 80%. The diameters of the extracted vibration region correspond to the increasing size of the fans in the captured images in Figure 9b.

### 3.4. Orientation

We analyzed the robustness of detection of the proposed vibration extraction method to changes in the orientation of the vibration source. For this experiment, several 512 × 512 videos of fans rotating at 37 rps were captured at 2000 fps from different orientations. The focal length, focus distance, and aperture were set to 50 mm, 4 m, and F5.0, respectively. The measurement area was 600 × 600 mm for 512 × 512 pixels at a distance of 5 m in front of the camera head, where one pixel corresponds to 1.2 mm${}^{2}$. The fan was mounted on a goniometer to measure its rotation degree, and was located 4 m in front of the camera. The initial rotation plane was 0${}^{\circ }$ with respect to the camera axis, and the angle was gradually increased to 90${}^{\circ }$ at intervals of 5${}^{\circ }$.

**Figure 10:**
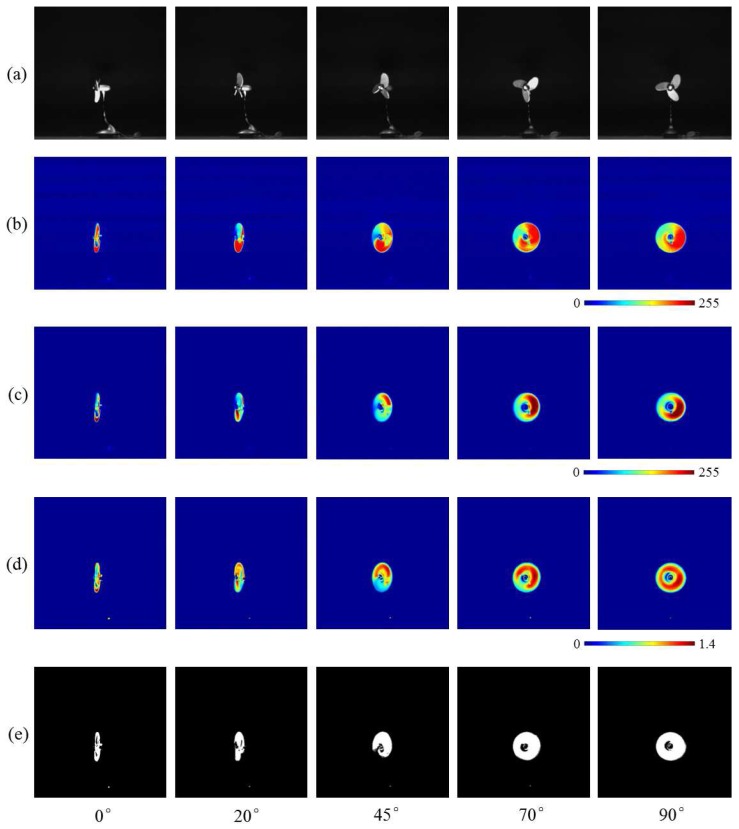
(**a**) Input images; amplitude of (**b**) input; and (**c**) pixel-wise filtered images; (**d**) amplitude ratios; (**e**) extracted vibration features.

Figure 10a shows the input 512 × 512 images at different orientations towards the camera lens. Figure 10b,c show the moving average distributions of amplitude of the input images and pixel-wise filtered images, respectively. Figure 10d shows the two amplitudes’ ratio distributions, and Figure 10e shows the extracted vibration regions.

**Figure 11:**
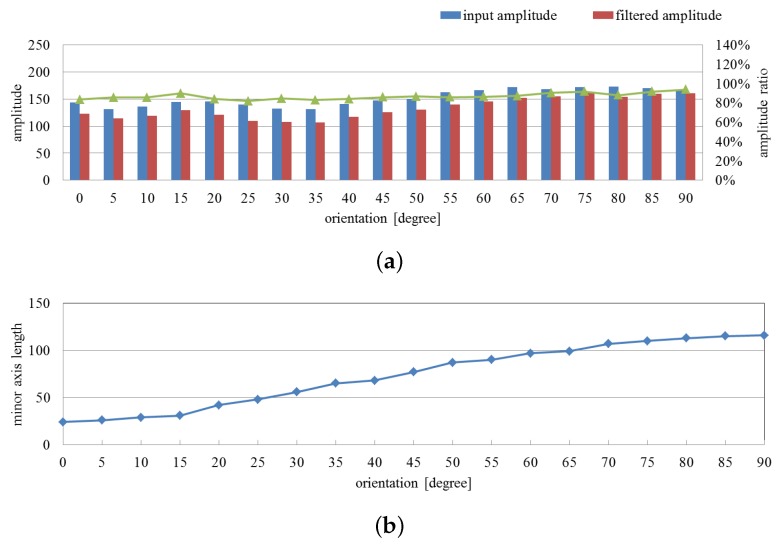
Averaged amplitude values and extracted region sizes with orientation variation. (**a**) Averaged amplitudes of input and pixel-wise filtered images and their ratios on the extracted pixels; (**b**) minor axis lengths of extracted vibration region.

The averages of the input and filtered images’ amplitude and their ratio on the extracted pixels are shown in Figure 11a, and the minor axis tendency of the extracted vibration region is shown in Figure 11b. From these figures, we can observe that the two amplitudes changed slightly with the rotation, whereas the ratio values remained relatively stable at around 85%. The minor axis of the extracted vibration region corresponds to the size of the fan in the captured images throughout the process.

### 3.5. Rotation Speed

We analyzed the frequency range of the proposed vibration extraction method by caturing several 512 × 512 videos of rotating fans at 2000 fps with different rotation speeds. The three fans used in this experiment were as described in Section 3.1; the rotation speed of the center fan was gradually increased from 26 rps to 44 rps in intervals of 1 rps, whereas those of the fans on the left and right were fixed at 44 rps and 26 rps, respectively. The distance from the camera to the object was 5 m. The focal length and aperture value were fixed at 50 mm and F1.4, respectively. The measurement area was 790 × 790 mm for 512 × 512 pixels at a distance of 5 m in front of the camera. The tap of coefficients and other parameters of the pixel-level band-pass filters were the same those in Section 3.1; their center frequencies and half-widths were 110 Hz and 10 Hz, respectively.

**Figure 12:**
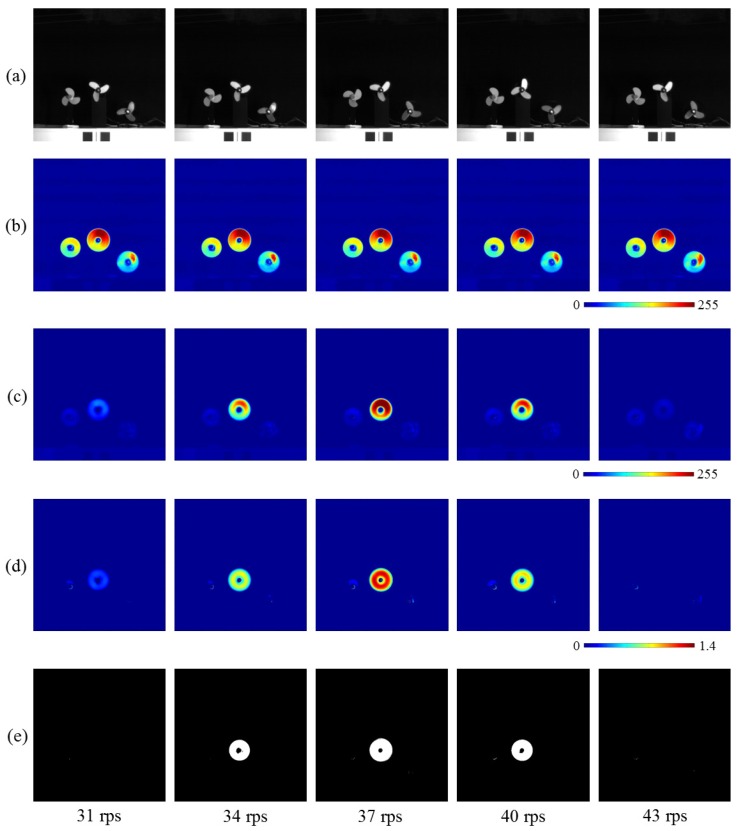
(**a**) Input images; amplitude of (**b**) input; and (**c**) pixel-wise filtered images; (**d**) amplitude ratios; (**e**) extracted vibration features.

Figure 12a shows the 512 × 512 input images with different rotation speeds from 31 rps to 43 rps. Figure 12b,c show the moving average distributions of amplitude of the input images and pixel-wise filtered images, respectively. Although the variation of the amplitudes of the input images was small in relation to the rotation speed, those of the extracted pixels around the center three-wing fan became greater when its rotation speed approached 37 rps, whose triple frequency almost corresponds to the center frequency 110 Hz of the band-pass filters. Figure 12d shows the ratio distributions of the two amplitudes, and Figure 12e shows the extracted vibration regions.

**Figure 13:**
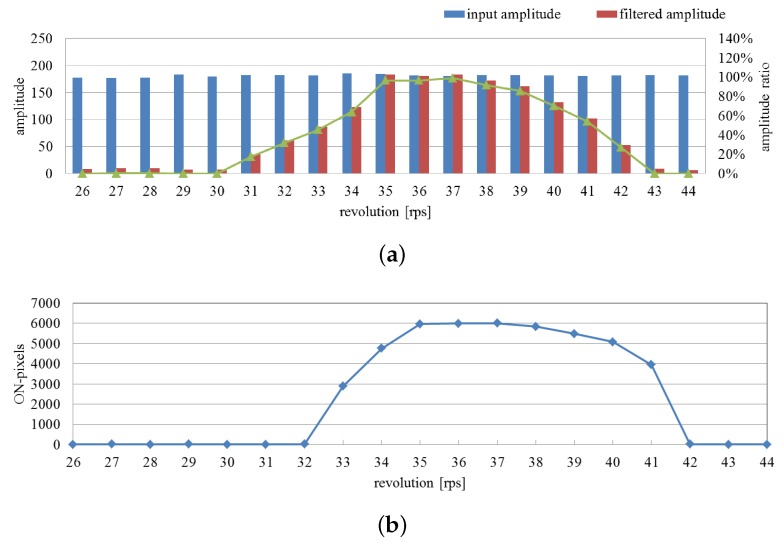
Averaged amplitude values and number of extracted pixels with rotation speed variation. (**a**) Averaged amplitudes of input and pixel-wise filtered images and their ratios; (**b**) number of extracted pixels as vibration region.

The average amplitude of the input and filtered images and their ratio on the specified pixels around the center fan are shown in Figure 13a when the rotation speed of the center fan was changed from 26 to 44 rps; the brightness was periodically changed from 78 to 132 Hz, according to the three wings of the fan. Here the specified pixels around the center fan were set to equal those of the extracted ones when the rotation speed was 37 rps. The number of the extracted pixels as vibration regions is shown in Figure 13b. Thus, the pixels around the center fan were distinctly extracted as vibration regions when its rotation speed was within 33 rps from 41 rps, which corresponds to the brightness changes in the frequency range from 99 to 123 Hz. It highly corresponds to the center frequency of 100 Hz and the half-width of 10 Hz of the pixel-level band-pass filters used in this experiment.

### 3.6. Moving Fan

**Figure 14:**
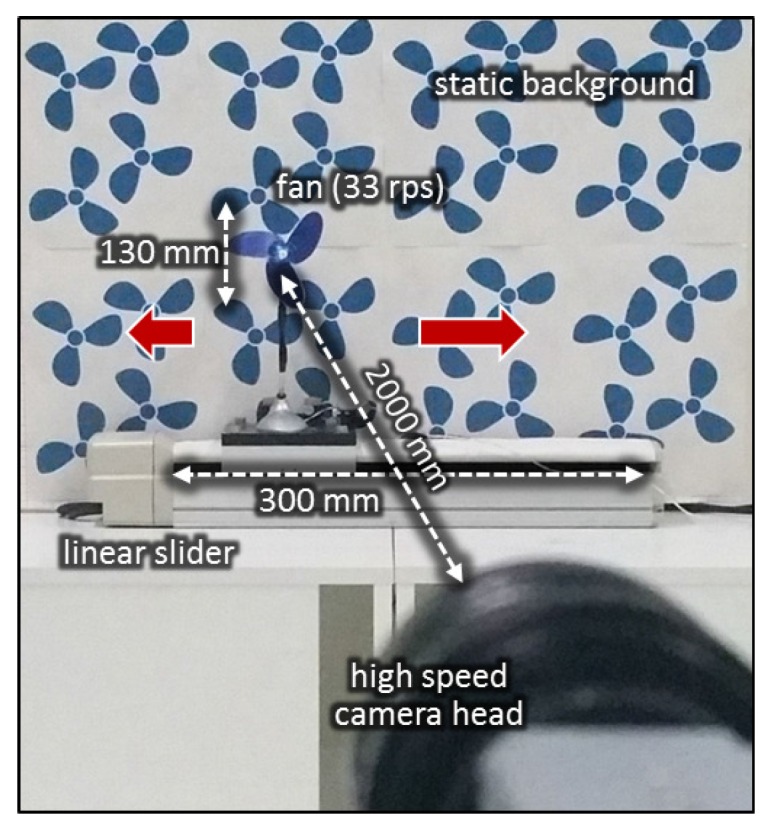
Moving fan against three-blades-patterned background.

We analyzed the robustness of the proposed vibration extraction method when a rotating fan moves against a complicated background scene. We captured 512 × 512 videos of a moving rotating fan for 1.5 s at 2000 fps with the environment illustrated in Figure 14. A 37-rps-rotation fan, whose size and rotation speed was the same as those used in Section 3.1, was installed on a linear slider. The distance from the camera head to the fan was 2 m. By controlling the slider mechanically, the fan moved alternatively in the right and left directions with an amplitude of 30 cm at a cycle of 1.5 s. A wallpaper patterned with many three-blade propellers, whose shape, size, and color were the same as those of the rotating fan, was used as a spatial jamming pattern in this experiment, because it is very difficult to distinguish the rotating fan from these patterns in a single image. The focal length and aperture value of the lens were 25 mm and F1.4, respectively. The measurement area was 500 × 500 mm${}^{2}$ for 512 × 512 pixels at a distance of 2 m in front of the camera, where one pixel corresponds to 1 mm${}^{2}$.

**Figure 15:**
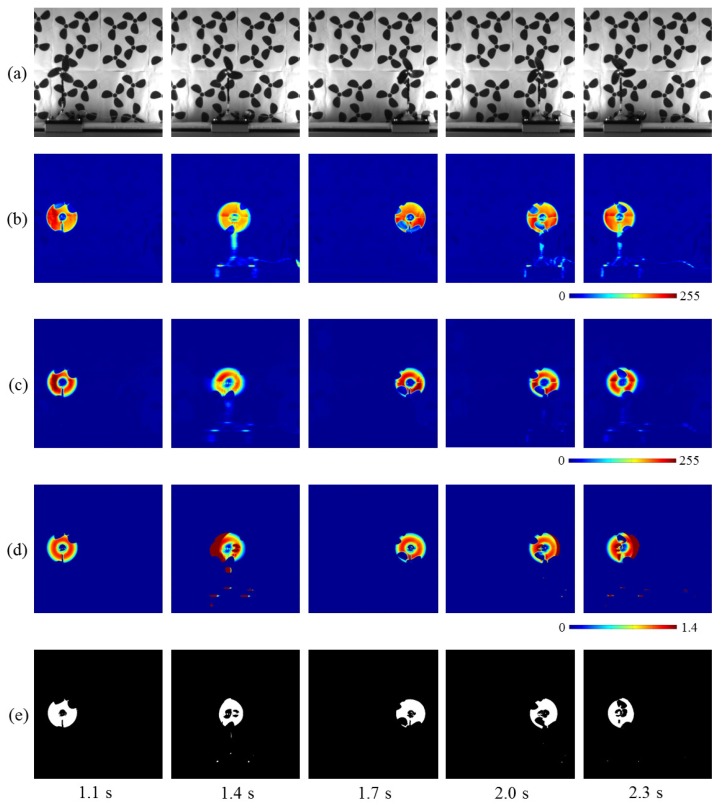
(**a**) Input images; amplitude of (**b**) input; and (**c**) pixel-wise filtered images; (**d**) amplitude ratios; (**e**) extracted vibration features.

Figure 15a shows the input of 512 × 512 images for 1.2 s, taken at intervals of 0.3 s. The translation speeds of the fan were 0.00, 0.96, 0.00, −0.40, and −0.60 m/s at time t= 1.1, 1.4, 1.7, 2.0, and 2.3 s, respectively; the positive/negative signs indicate the movements in the right/left direction. Figure 15b,c show the moving average distributions of the amplitude of the input and pixel-wise filtered images, respectively. Both the moving average values in (b) and (c) became larger at the pixels around the moving fan, whereas the moving average distributions of the pixel-wise filtered images were slightly dilated in the direction opposite to the movement direction of the fan, because of the latency effect in the digital filter. Figure 15d shows the two amplitudes’ ratio distributions, and Figure 15e shows the extracted vibration regions. These regions excluded the pixels around the three-blade-fan patterns on the background wallpaper, and they only involved those around the moving fan. Several pixels around the fan were not detected, because of the close similarity of the brightness around its blades with that of the background three-blade-patterns. Thus, the brightness changed very little with time when the fan was passing over the background patterns.

**Figure 16:**
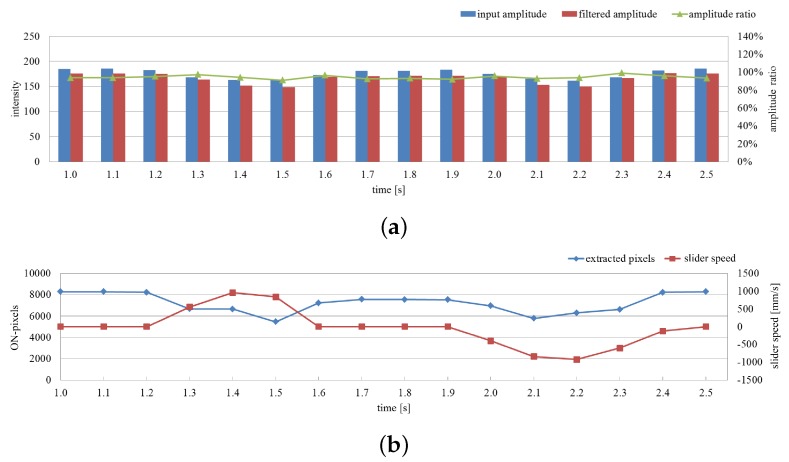
Averaged amplitude values and number of extracted pixels when a rotating fan moves. (**a**) Averaged amplitudes of input and pixel-wise filtered images and their ratios on the extracted pixels; (**b**) number of extracted pixels as vibration region and slider speeds.

The average amplitude of the input and filtered images and their ratio on the extracted pixels are shown in Figure 16a for 1.5 s, and the number of extracted pixels as vibration regions and the translation speeds of the fan are shown in Figure 16b. When the rotating fan was moving alternatively in the right and left directions, the ratio remained at around 90% whereas the two amplitudes slightly changed. Here the number of extracted pixels decreased around t= 1.5 and 2.1 s when the translation speed of the fan increased. Because of the latency effect in the digital filter; the vibration features were not extracted at the pixels around the side of the rotating fan opposite to its movement direction as illustrated in Figure 15e. Nevertheless, these results apparently indicate the robustness of the proposed vibration extraction method when a rotating fan moves against a complicated background.

### 3.7. Moving Background

We analyzed the robustness of the proposed vibration source extraction method when observing a rotating fan against a moving background scene. The experimental setting, which includes the distance from the camera to the fan, the lens parameters, the background pattern, and the moving speed of the linear slider, was similar as that used in Section 3.6, except that the 37-rps-rotating fan was fixed and the three-blades-patterned wallpaper was installed on a linear slider to enable the background wallpaper to move in the right and left directions at a cycle time of 1.5 s.

**Figure 17:**
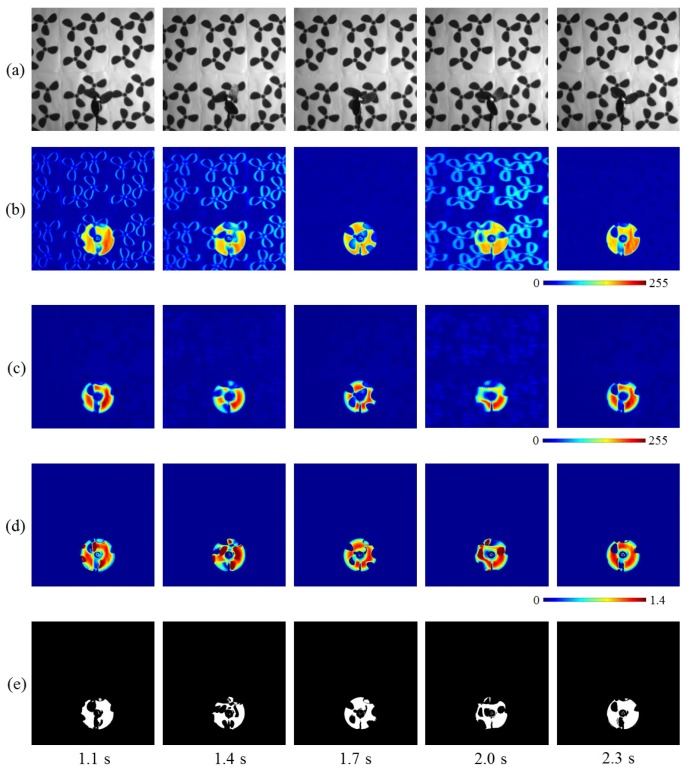
(**a**) Input images; amplitude of (**b**) input; and (**c**) pixel-wise filtered images; (**d**) amplitude ratios; (**e**) extracted vibration features.

Figure 17a shows the input 512 × 512 images. The background moved at speeds of 0.32, 0.64, 0.00, −0.8, and 0.00 m/s at time t= 1.1, 1.4, 1.7, 2.0, and 2.3 s, respectively. Figure 17b,c show the moving average distributions of the amplitude of the input and pixel-wise filtered images, respectively. Due to the movement of the background wallpaper, the moving averages in (b) had certain values at the pixels around the edges of the three-blades-patterns, whereas those in (c) became larger only at the pixels around the rotating fan. Figure 17d shows the ratio distributions of the two amplitudes, and Figure 17e shows the extracted vibration regions. The extracted regions did not include the pixels around the edges of the three-blades-patterns, and they involved only the pixels around the fan. This means that its neighboring pixels were not always detected for the same reason described in Section 3.6.

**Figure 18:**
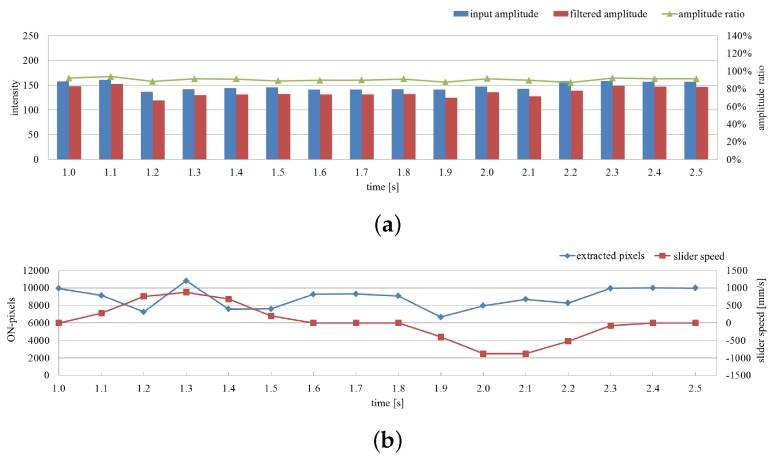
Averaged amplitude values and number of extracted pixels with moving background. (**a**) Averaged amplitudes of input and pixel-wise filtered images and their ratios on the extracted pixels; (**b**) number of extracted pixels as vibration region and slider speeds.

The average amplitude of the input and filtered images and their ratio on the extracted pixels are shown in Figure 18a for 1.5 s, and the number of extracted pixels as vibration regions and the speeds of background wallpaper are shown in Figure 18b. The two amplitudes slightly fluctuated, whereas the ratio remained at around 90% when the background wallpaper was moving alternatively in the right and left directions. The number of extracted pixels slightly fluctuated because several pixels around the rotating fan were not extracted as illustrated in Figure 17e, where the blades of the fan and the moving three-blades-patterns overlapped. Nevertheless, these results apparently indicate the robustness of the proposed vibration extraction method for a rotating fan against a moving patterned background.

## 4. Experiment for a Flying Multicopter

We analyzed the robustness of our vibration source tracking method with a flying multicopter in two non-controlled outdoor scenarios where additional distraction moving objects and unstructured backgrounds were presented; (a) trees-and-building background; and (b) walking-persons background. In the experiments, we examined that the simultaneous effect of the multiple appearance variations tested in the previous section robustly functions in real scenarios with cluttered and moving backgrounds. The multicopter used in the experiments was an RC EYE One Xtreme (CEI Conrad Electronic Intl. (HK) Ltd., Hong Kong, China) with four 138-mm dual-blade propellers. The multicopter had dimensions of 225 × 225 × 80 cm, excluding propellers. The flapping frequency of each propeller varied within the range 80–100 Hz according to the flight operation commands. Color 512 × 512 videos of a flying multicopter were captured offline at 1000 fps for 15 s in each scenario with the recording time being limited by the memory size of the high-speed camera. The body and propellers of the multicopter were painted red to extract its location in images for evaluation, whereas our algorithm was processed for gray-level images. In the experiments, the tap coefficients $a_{s}$, $b_{s}$ of the pixel-level digital filters were set to operate as band-pass filters with a center frequency of $f_{0}=$ 80 Hz (twice the flapping frequency of the dual-blade propellers) and half-width of 20 Hz. The other parameters were set to p=4, $\Delta T_{f}=$ 44 m·s, and $T_{0}=1/f_{0}=6$ m·s. The thresholds $\theta_{1}$ and $\theta_{2}$ were set to 20 and 0.5, respectively.

### 4.1. Trees-and-Building Background

We analyzed the 1000-fps video when the multicopter moves against an unstructured background. The multicopter flew in the right and left directions with vertical elevation twice in 15 s in front of trees and a building, which were located at a distance of approximately 8 m in front of the camera. The focal length, focus distance, and aperture of the lens were set to 12 mm, 8 m, and F2.8, respectively. The measurement areas of 512 × 512 pixels were 5.3 × 5.3 m, where one pixel corresponds to 10.3 mm${}^{2}$ at a distance of 8 m.

**Figure 19:**
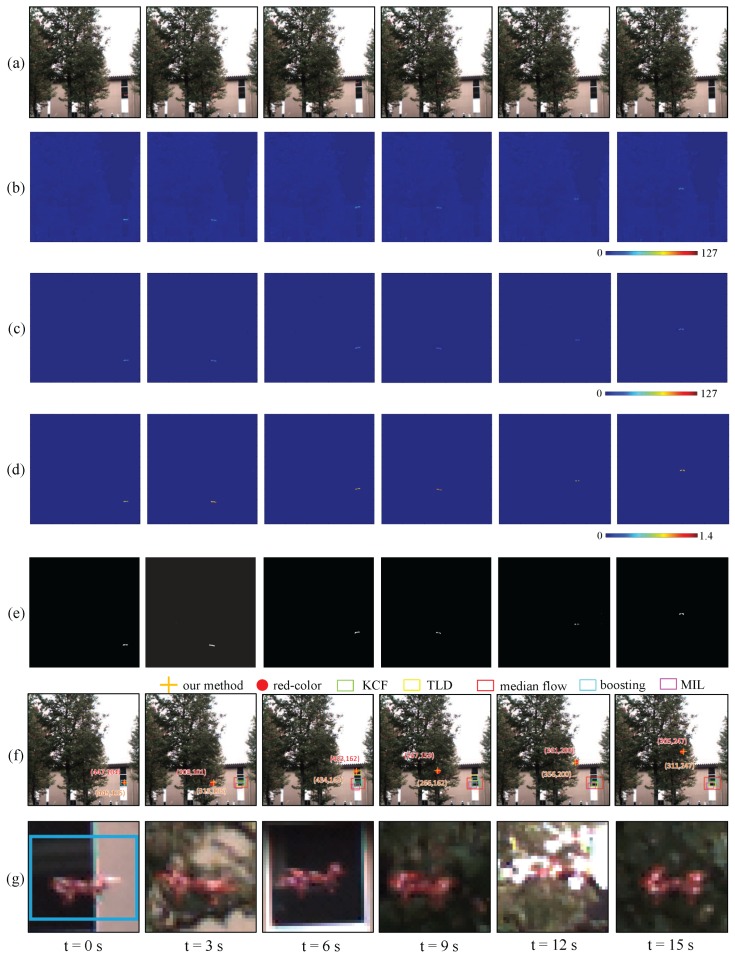
(**a**) Input images; amplitude of (**b**) input; and (**c**) pixel-wise filtered images; (**d**) amplitude ratios; (**e**) extracted vibration features; (**f**) tracked positions; (**g**) magnified images.

Figure 19a–d shows the input images and the moving average distributions of the amplitude of the input images and pixel-wise filtered images, as well as the ratio distribution of the two amplitudes’. The images were taken at intervals of 3 s for t= 0–15 s. Figure 19e,f show the vibration regions extracted by our algorithm, and magnified images of 32 × 32 pixels around the averaged positions of the extracted pixels, respectively. These averaged positions (blue “+” s) were plotted over the input images as well as those of the red-color regions (red “+” s) in Figure 19g; they corresponded to the locations of the red multicopter in images. For comparison, the tracking results of the other appearance-based single-object tracking methods, which were prepared in Open CV Tracking API in Open CV 3.0 [67], were illustrated as color-lined rectangular regions; (1) KCF [68]; (2) TLD [69]; (3) Median Flow [70]; (4) Boosting [71]; and (5) MIL [72]. The color input images at 1000 fps were processed for all the single-object tracking methods, and the object to be tracked was initially defined as the 32 × 24 subimage in the 32 × 32 ROI region at t= 0 s as illustrated in Figure 19f.

It can be seen that certain pixels around the propellers of the multicopter were robustly extracted as vibration features by our algorithm when the background scene just directly behind the multicopter was varying with its flight trajectory (trees at t= 3, 9, 12, and 15 s, and building at t= 0 and 6 s). When t=0, 3, 6, 9, 12, and 15 s, the averaged positions of the red-color regions in the images, which indicated to the actual locations of the multicopter, were (447,104), (308,101), (432,162), (267,159), (361,200), and (305,247), respectively, whereas those of the extracted pixels were (445,105), (313,106), (434,163), (266,162), (356,200), and (312,247), respectively. Due to the partial occlusion of the propellers by the multicopter itself, the averaged positions of the extracted pixels slightly deviated from the actual locations of the multicopter, however, they almost corresponded with the actual locations of the multicopter and the ROI regions illustrated in Figure 19f wholly or partially involved the regions of the multicopter. In Figure 19g, it can be seen that the tracking windows largely deviated from the target multicopter and mistracked cluttered background scenes in all the single-object tracking methods. This is because the object to be tracked was determined with a subimage in the low-resolution 32 × 24 region, and there were many unstructured patterns with similar appearance-based features in the background scenes.

**Figure 20:**
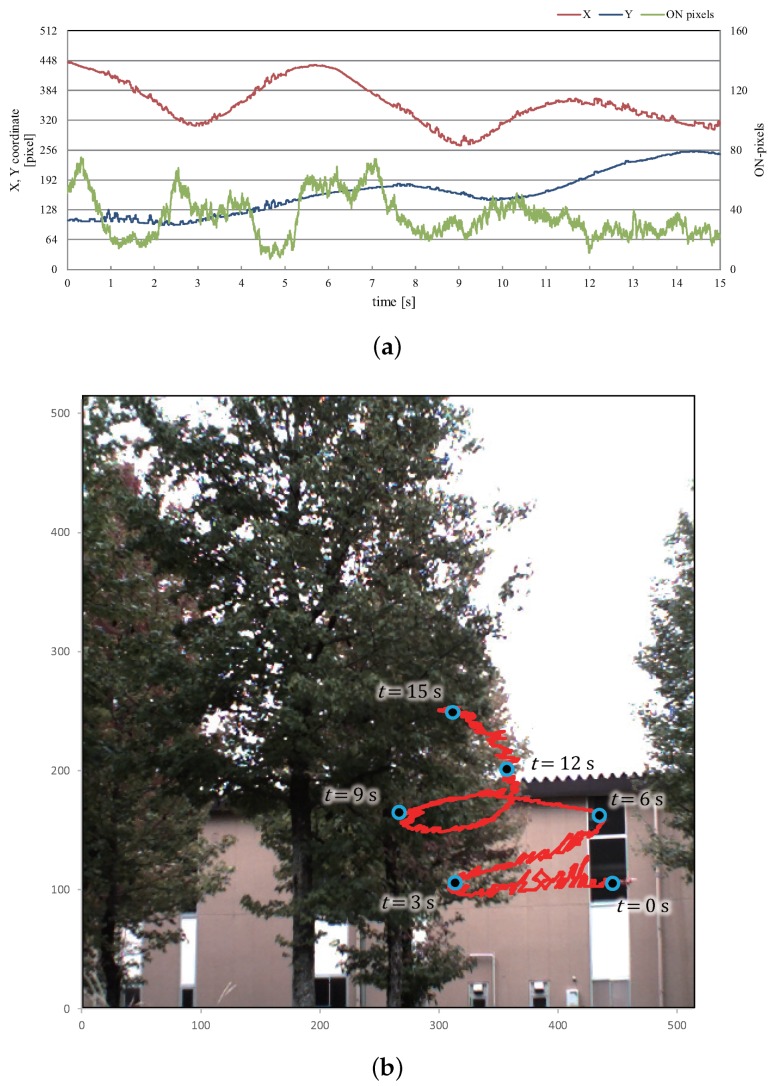
xy trajectory of extracted vibration region in “trees-and-building background” experiment. (**a**) *x*- and *y*-coordinates and number of pixels; (**b**) xy trajectory.

Figure 20a illustrates graphs that show changes in the *x*- and *y*-coordinate values of the averaged positions of the extracted pixels and the number of the extracted pixels for 15 s, and the xy trajectory for 15 s was plotted over the input image of 512 × 512 pixels captured at t=0 in Figure 20b. Whereas the number of the extracted pixels was not so large and varied in the range of 7 to 75, we have confirmed that the xy trajectory of the averaged positions of the extracted pixels were robustly extracted in correspondence with the left-and-right motion and elevation of the flying multicopter when the background scene directly behind the multicopter was frequently switched to trees in the center and a building in the right side. Here we can observe certain fluctuations in the xy trajectory due to the partial occlusion of the propellers. This is because our method only extracted the regions of the propellers, by excluding the body of the multicopter, and the average positions of the extracted pixels were discretely changed within the region of the multicopter when one propeller was unobservable with occlusion.

### 4.2. Walking-Persons Background

We analyzed the 1000-fps video when the multicopter moves against a background with moving obstacles; the multicopter flew repeatedly in the right and left directions at different heights in front of many persons with quick arm movements, who were walking at a distance of approximately 6 m in front of the camera. The focal length, focus distance, and aperture of the lens were set to 12 mm, 8 m, and F2.8, respectively. The measurement areas of 512 × 512 pixels were 4.7 × 4.7 m, where one pixel corresponds to 9.2 mm${}^{2}$ at a distance of 6 m.

**Figure 21:**
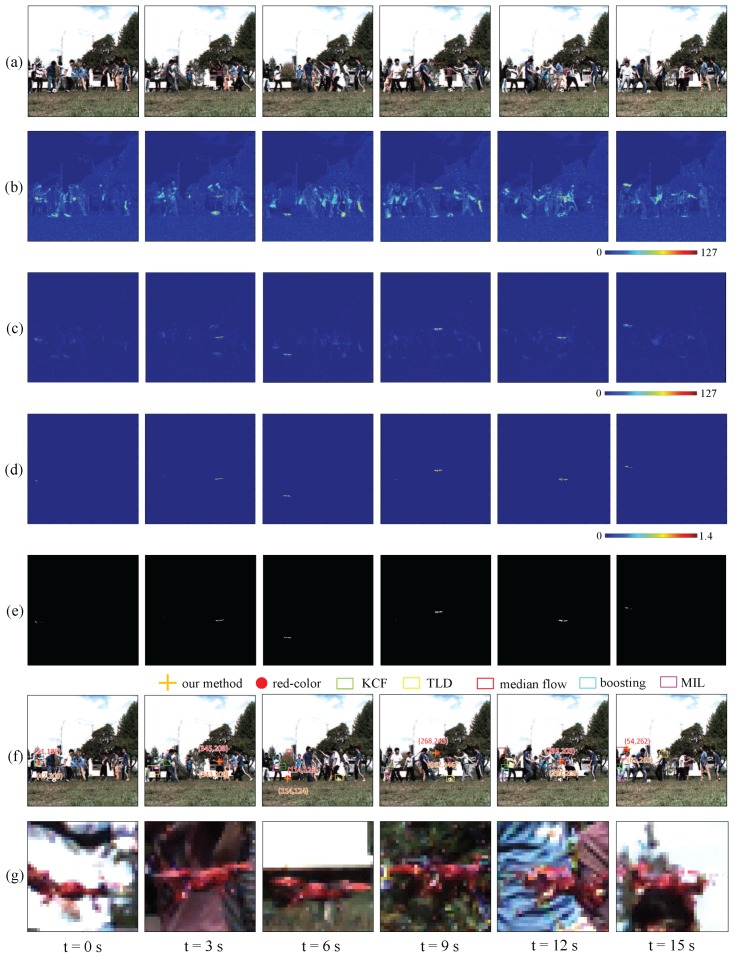
(**a**) Input images; amplitude of (**b**) input; and (**c**) pixel-wise filtered images; (**d**) amplitude ratios; (**e**) extracted vibration features; (**f**) tracked positions; (**g**) magnified images.

Figure 21a–d shows the input images, the moving average distributions of the amplitude of the input images, pixel-wise filtered images, and the ratio distribution of the two amplitudes for t= 0–15 s. Figure 21e,f show the vibration regions extracted by our algorithm, and magnified images of 32 × 32 pixels around the extracted pixels, respectively. Figure 21g shows the averaged positions of the extracted pixels, those of the red-color regions, and the tracking results of the single object tracking methods used in the previous subsection, in which the object to be tracked was initially defined as a 32 × 24 subimage at t= 0 s as illustrated in Figure 21f. When the multicopter flew repeatedly in the right and left directions at different heights in front of many walking persons, our algorithm extracted certain pixels around the propellers of the multicopter as vibration features without being disturbed by their quick movements. When t=0, 3, 6, 9, 12, and 15 s, the averaged positions of the red-color regions in the images were (51,199), (345,208), (114,128), (268,245), (295,205), and (54,262), respectively, and those of the extracted pixels, (47,200), (343,209), (114,124), (262,246), (291,208), and (61,265), respectively, had slight deviations from them due to the partial occlusion of the propellers, however, the ROI regions illustrated in Figure 21f involved the regions of the multicopter at all times. Figure 21g shows that the tracking windows with the single-object tracking methods, which were used in the previous subsection, largely deviated from the target mulitcopter, and these appearance-based tracking methods are almost unable to track in this scenario.

**Figure 22:**
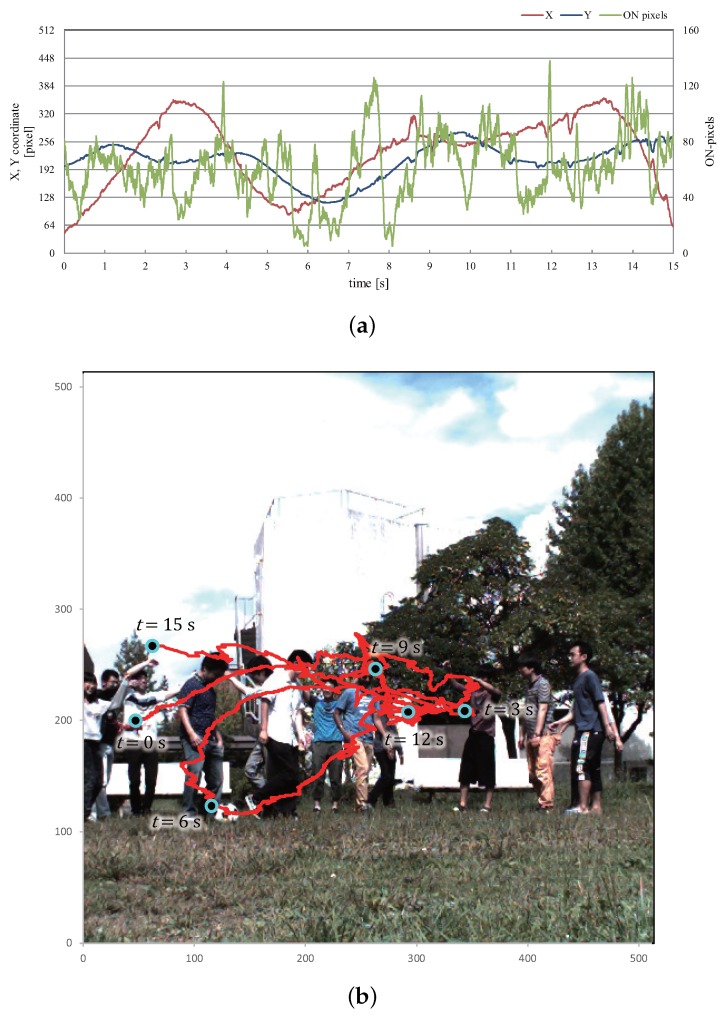
xy trajectory of extracted vibration region in “trees-and-building background” experiment. (**a**) *x*- and *y*-coordinates and number of pixels; (**b**) xy trajectory.

Figure 22a,b illustrate graphs that show changes in the *x*- and *y*-coordinate values of the averaged positions of the extracted pixels and the number of extracted pixels for 15 s, and the xy trajectory for 15 s was plotted over the input image at t=0. Corresponding to the left-and-right motion of the flying multicopter at different heights, the xy trajectory of the averaged positions of the extracted pixels were robustly extracted without any disturbance by the moving background, including the fluctuation due to the partial occlusion of the propellers, whereas the number of extracted pixels largely varied in the range of 5 to 138.

## 5. Conclusions and Future Work

In this paper, we analyzed the detectability of a vibration source localization method based on pixel-level digital filters applied to HFR video for rotating fans and a flying multicopter with rotating propellers under various imaging conditions, whose rotational frequencies were distinctly distant from those of the background scenes. The robustness of the method under brightness changes, defocus blur, apparent scale and pose variations, rotational frequency change, and complex background, was demonstrated using several 2000 fps videos of rotating fans captured by adjusting the lens parameters, the shooting angle, and the rotation of the fan or by moving the fan and background pattern. The robustness of images that were simultaneously affected by multiple appearance changes was also demonstrated using a flying multicopter in various outside scenarios.

This study concentrated on the primitive vibration source localization with pixel-level band-pass filters for temporal brightness changes, and it did not directly concern the geometric motion of a target object; the frequency range of temporal brightness changes at pixels around the target object may not be matched with that of its geometric motion when the target object has a periodic surface pattern. To realize a more universal vibration feature detector, which is invariant to any spatial appearance of the target object, it becomes more effective to apply our pixel-level band-pass filters to geometric motion fields estimated by optical flow [73,74]. This is one of well-known image processing algorithms, instead of using the image brightness. Besides, by combining our proposed dynamics-based vibration feature with appearance-based recognition methods, the accuracy and robustness in vibration source localization will be remarkably improved when the target frequency range overlaps with that of background scenes. Thus, in future work, we intend to improve these points toward more universal vibration source localization under more extreme conditions and accelerate the computational speed for real-time processing of HFR video, thus enabling practical applications such as drone surveillance.

## Figures and Tables

**Table 1:** Execution times on PC.

**Image Size**	64 × 64	128 × 128	256 × 256	512 × 512	1024 × 1024	2048 × 2048
**Exec Time**	0.16 ms	0.66 ms	2.69 ms	10.47 ms	39.78 ms	157.38 ms
